# Minutes of the Proceedings of the Virginia Society of Dental Surgeons, Together with the Constitution and By-Laws, Adopted at Its Organization, December 12, 1842

**Published:** 1843-03

**Authors:** James D. McCabe, Jno. Geo. Wayt, R. N. Hudson

**Affiliations:** Ch'n.


*Minutes of the Proceedings of the Virginia Society of Dental Surgeons, together with the Constitution and By-laws, adopted at its organization, December* 12, 1842.

The above is the title of a pamphlet of 21 pages, very neatly gotten up, which we have received from Dr. James D. McCabe, corresponding and recording secretary of the Virginia Society of Dental Surgeons. We published in our last number the minutes of the proceedings of this society, so that further notice of them is unnecessary at this time. But connected with the proceedings and constitution and by-laws, is a well-written and excellent address, prepared by Dr. J. D. McCabe, chairman of a committee appointed for the purpose, which we take the liberty of copying.—Eds.

*To the Members of the Dental Profession in Virginia:*

Gentlemen:—The undersigned, a Committee appointed by "The Virginia Society of Dental Surgeons," respectfully request permission to call your attention to the organization of that Society, and ask your co-operation in the furtherance of its views for the advancement of the interests of the profession in our State.

With us you have no doubt long mourned the various evils under which Dental Surgery has labored, occasioned by the lack of some common centre, from which should radiate the elements of a sound Dental education, or the means of detecting quackery and charlatanism, and stamping upon merit the evidence of proper application to the rules of our art. This long night was at length broken by the institution of "The American Society of Dental Surgeons." To this succeeded the Baltimore College of Dental Surgery, an institution which merits and should receive the fostering care and support of every member of the profession. These have already done much for our art, in furnishing a bond of union to those who prefer honest fame to gold, and establishing a sound system of professional education. Last but not least, the Virginia Society has entered the arena, to accomplish in our own State what has been so successfully effected north of us. What it is this last Society desires to effect, will be seen by reference to the accompanying Constitution and By-Laws.

Too long has our favorite science been considered a mere mechanical trade, into which every unprepared quack, acquainted with the use of small tools, might obtrude himself at pleasure. Men of high attainments in other branches of the healing art, have lent themselves to this delusion, and greatly contributed to the injury of a science, that ranks deservedly high, when well understood and practised, and aids in effecting as much for the relief of human suffering, as any one branch of the healing art.

It is time that the virtue and intelligence of the profession in Virginia, should unite to root away its reproaches, and erect a standard of qualification, which will place Dental Surgery where it should be, alongside its sister sciences, Medicine and General Surgery.

The Virginia Society aims to effect in this State a consummation so much desired, and so devoutly wished for by us all: this it hopes to do by a rigid adherence to the requisites for membership in its body, and thus to unite all who respect their profession, in an effort to protect it from the unhallowed hands of the ignorant and pretending, as well as to secure that professional unity and courtesy, which is so essential to the improvement and elevation of the science.

With these views to govern our undertaking, we call to our aid our professional brethren; those who may wish to unite with us, will please forward their application to the Examining Committee, or any one of them, and give us their attendance at the annual meeting, to be held in the City of Richmond, on the second Wednesday in October next.

We remain gentlemen, your ob't servants,

JAMES D. McCABE, *Ch'n.*
JNO. GEO. WAYT,
R. N. HUDSON.

### Miscellaneous Notices.

